# 1-Benzyl­idene­amino-3-(4-methyl­phen­yl)thio­urea

**DOI:** 10.1107/S160053681100198X

**Published:** 2011-01-22

**Authors:** Yan-Ling Zhang, Zhi-Hong Xu, Fu-Juan Zhang, Feng-Ling Yang

**Affiliations:** a College of Chemistry and Chemical Engineering, Xuchang University, Xuchang, Henan Province 461000, People’s Republic of China

## Abstract

In the title compound, C_15_H_15_N_3_S, the almost planar 2-benzyl­idenehydrazinecarbothio­amide unit (r.m.s. deviation = 0.0351 Å) is aligned at a dihedral angle of 64.42 (6)° with respect to the plane of the tolyl ring. The mol­ecule exhibits an *E* configuration for the azomethine linkage. In the crystal, inter­molecular N—H⋯S hydrogen bonds about centers of inversion lead to the formation of dimers.

## Related literature

For biological applications of thio­semicarbazones, see: Hu *et al.* (2006 ▶).
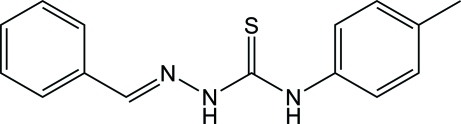


## Experimental

### 

#### Crystal data

C_15_H_15_N_3_S
*M* *_r_* = 269.36
Monoclinic, 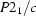

*a* = 10.2359 (3) Å
*b* = 16.0648 (3) Å
*c* = 9.9703 (3) Å
β = 117.154 (4)°
*V* = 1458.81 (7) Å^3^
*Z* = 4
Cu *K*α radiation
μ = 1.88 mm^−1^
*T* = 293 K
0.30 × 0.20 × 0.18 mm

#### Data collection

Oxford Diffraction Xcalibur Eos Gemini diffractometer
Absorption correction: multi-scan (*CrysAlis PRO*; Oxford Diffraction, 2010 ▶) *T*_min_ = 0.603, *T*_max_ = 0.729
12637 measured reflections
2605 independent reflections
2253 reflections with *I* > 2σ(*I*)
*R*_int_ = 0.026
Standard reflections: 0

#### Refinement

*R*[*F* ^2^ > 2σ(*F* ^2^)] = 0.041
*wR*(*F* ^2^) = 0.120
*S* = 1.05
2605 reflections
181 parameters
H atoms treated by a mixture of independent and constrained refinement
Δρ_max_ = 0.19 e Å^−3^
Δρ_min_ = −0.21 e Å^−3^

### 

Data collection: *CrysAlis PRO* (Oxford Diffraction, 2010 ▶); cell refinement: *CrysAlis PRO*; data reduction: *CrysAlis PRO*; program(s) used to solve structure: *SHELXS97* (Sheldrick, 2008 ▶); program(s) used to refine structure: *SHELXL97* (Sheldrick, 2008 ▶); molecular graphics: *OLEX2* (Dolomanov *et al.*, 2009 ▶); software used to prepare material for publication: *OLEX2*.

## Supplementary Material

Crystal structure: contains datablocks I, global. DOI: 10.1107/S160053681100198X/ng5104sup1.cif

Structure factors: contains datablocks I. DOI: 10.1107/S160053681100198X/ng5104Isup2.hkl

Additional supplementary materials:  crystallographic information; 3D view; checkCIF report

## Figures and Tables

**Table 1:** Hydrogen-bond geometry (Å, °)

*D*—H⋯*A*	*D*—H	H⋯*A*	*D*⋯*A*	*D*—H⋯*A*
N2—H2⋯S1^i^	0.88 (2)	2.48 (2)	3.3522 (15)	170.3 (17)

Symmetry code: (i) 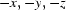.

**Fig. 1.**
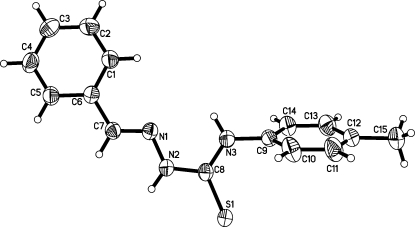
The molecular structure of the title compound, with displacement ellipsoids drawn at 30% probability level.

**Fig. 2.**
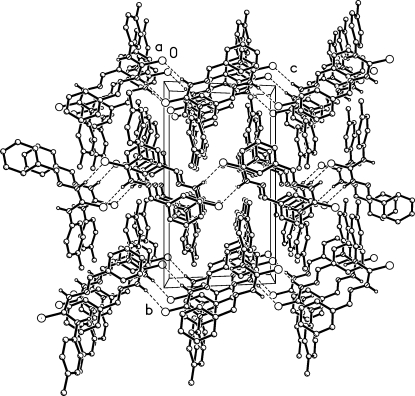
The packing diagram of the title compound. Intermolecular hydrogen bonds are shown as dashed line.

## supplementary crystallographic information

### Comment

Thiosemicarbazones have attracted our attention because of their biological applications (Hu *et al.*, 2006). A few single-crystal reports about them are presented. Detailed information on their molecular and crystal structures is necessary to understand their anticancer activity. The molecular structure of (I) is shown in Fig 1. The molecules exhibit an E configuration.The thiosemicarbazide and benzaldehyde unit are located on opposite sides of the N1=C7 bond.The 2-benzylidenehydrazinecarbothioamide unit has a planar configuration and subtends an angle of 64.42 (6)° with respect to the plane of the tolyl ring.In the crystal structure of the title compound, there is N(2)—H(2)···S(1)#1 hydrogen-bond interactions in molecules which leads to a supramolecular architecture (Fig. 2).

### Experimental

*N*-(*p*-Tolyl)hydrazinecarbothioamide (2.7 g,15 mmol) and benzaldehyde (1.6 g, 15 mmol) was dissolved in 95% ethanol (20 ml) and the solution was refluxed for 6.5 h. Fine colorless crystals appeared on cooling. They were filtered and washed by 95% ethanol to give 2.6 g of the title compound in 65% yield. Single crystals suitable for X-ray measurements were obtained from 2-propanol by slow evaporation at room temperature.

### Refinement

H atoms were placed in calculated positions with C—H = 0.93–0.96 and N—H = 0.88–0.90 Å, and refined using a riding model, *U*_iso_(H) = 1.5*U*_eq_(C) for methyl H atoms and 1.2*U*_eq_(C,*N*).

### Crystal data

**Table:**

C_15_H_15_N_3_S	*F*(000) = 568
*M**_r_* = 269.36	*D*_x_ = 1.226 Mg m^−^^3^
Monoclinic, *P*2_1_/*c*	Cu *K*α radiation, λ = 1.54184 Å
Hall symbol: -P 2ybc	Cell parameters from 7009 reflections
*a* = 10.2359 (3) Å	θ = 4.9–72.1°
*b* = 16.0648 (3) Å	µ = 1.88 mm^−^^1^
*c* = 9.9703 (3) Å	*T* = 293 K
β = 117.154 (4)°	Prismatic, colorless
*V* = 1458.81 (7) Å^3^	0.30 × 0.20 × 0.18 mm
*Z* = 4	

### Data collection

**Table:**

Oxford Diffraction Xcalibur Eos Gemini diffractometer	2605 independent reflections
Radiation source: fine-focus sealed tube	2253 reflections with *I* > 2σ(*I*)
graphite	*R*_int_ = 0.026
ω scans	θ_max_ = 67.1°, θ_min_ = 4.9°
Absorption correction: multi-scan (*CrysAlis PRO*; Oxford Diffraction, 2010)	*h* = −12→12
*T*_min_ = 0.603, *T*_max_ = 0.729	*k* = −19→19
12637 measured reflections	*l* = −8→11

### Refinement

**Table:**

Refinement on *F*^2^	Primary atom site location: structure-invariant direct methods
Least-squares matrix: full	Secondary atom site location: difference Fourier map
*R*[*F*^2^ > 2σ(*F*^2^)] = 0.041	Hydrogen site location: inferred from neighbouring sites
*wR*(*F*^2^) = 0.120	H atoms treated by a mixture of independent and constrained refinement
*S* = 1.05	*w* = 1/[σ^2^(*F*_o_^2^) + (0.0727*P*)^2^ + 0.1569*P*] where *P* = (*F*_o_^2^ + 2*F*_c_^2^)/3
2605 reflections	(Δ/σ)_max_ < 0.001
181 parameters	Δρ_max_ = 0.19 e Å^−^^3^
0 restraints	Δρ_min_ = −0.21 e Å^−^^3^

### Special details

**Table:**

Geometry. All e.s.d.'s (except the e.s.d. in the dihedral angle between two l.s. planes) are estimated using the full covariance matrix. The cell e.s.d.'s are taken into account individually in the estimation of e.s.d.'s in distances, angles and torsion angles; correlations between e.s.d.'s in cell parameters are only used when they are defined by crystal symmetry. An approximate (isotropic) treatment of cell e.s.d.'s is used for estimating e.s.d.'s involving l.s. planes.
Refinement. Refinement of *F*^2^ against ALL reflections. The weighted *R*-factor *wR* and goodness of fit *S* are based on *F*^2^, conventional *R*-factors *R* are based on *F*, with *F* set to zero for negative *F*^2^. The threshold expression of *F*^2^ > σ(*F*^2^) is used only for calculating *R*-factors(gt) *etc*. and is not relevant to the choice of reflections for refinement. *R*-factors based on *F*^2^ are statistically about twice as large as those based on *F*, and *R*- factors based on ALL data will be even larger.

### Fractional atomic coordinates and isotropic or equivalent isotropic displacement parameters (Å^2^)

**Table:**

	*x*	*y*	*z*	*U*_iso_*/*U*_eq_	
S1	0.13263 (6)	0.10810 (3)	0.02008 (5)	0.0727 (2)	
N1	0.15900 (15)	−0.00305 (8)	0.37897 (15)	0.0546 (3)	
N2	0.12114 (16)	0.01805 (8)	0.23296 (16)	0.0566 (3)	
N3	0.28187 (16)	0.12678 (9)	0.31818 (17)	0.0599 (4)	
C1	0.2093 (2)	−0.05346 (12)	0.6752 (2)	0.0684 (5)	
H1	0.2567	−0.0052	0.6694	0.082*	
C2	0.2298 (3)	−0.08202 (15)	0.8131 (2)	0.0815 (6)	
H2A	0.2905	−0.0528	0.9000	0.098*	
C3	0.1610 (3)	−0.15388 (14)	0.8239 (2)	0.0798 (6)	
H3A	0.1763	−0.1736	0.9177	0.096*	
C4	0.0699 (3)	−0.19596 (13)	0.6949 (2)	0.0765 (5)	
H4	0.0226	−0.2441	0.7014	0.092*	
C5	0.0481 (2)	−0.16751 (11)	0.5560 (2)	0.0667 (4)	
H5	−0.0140	−0.1964	0.4693	0.080*	
C6	0.11855 (18)	−0.09582 (10)	0.5445 (2)	0.0560 (4)	
C7	0.09253 (18)	−0.06621 (10)	0.39603 (19)	0.0569 (4)	
H7	0.0252	−0.0943	0.3110	0.068*	
C8	0.18293 (17)	0.08400 (9)	0.20078 (19)	0.0532 (4)	
C9	0.35959 (17)	0.19777 (10)	0.30466 (18)	0.0542 (4)	
C10	0.4599 (2)	0.18975 (13)	0.2504 (3)	0.0838 (6)	
H10	0.4801	0.1375	0.2241	0.101*	
C11	0.5311 (3)	0.25904 (16)	0.2346 (3)	0.0938 (8)	
H11	0.5983	0.2529	0.1965	0.113*	
C12	0.5053 (2)	0.33670 (13)	0.2737 (2)	0.0750 (5)	
C13	0.4092 (2)	0.34307 (11)	0.3338 (3)	0.0790 (5)	
H13	0.3933	0.3948	0.3656	0.095*	
C14	0.3353 (2)	0.27444 (11)	0.3483 (2)	0.0670 (5)	
H14	0.2692	0.2804	0.3877	0.080*	
C15	0.5825 (3)	0.41238 (18)	0.2529 (3)	0.1188 (11)	
H15B	0.6778	0.3966	0.2653	0.178*	
H15C	0.5928	0.4538	0.3265	0.178*	
H15A	0.5257	0.4347	0.1536	0.178*	
H2	0.054 (2)	−0.0105 (12)	0.158 (2)	0.067 (5)*	
H3	0.294 (2)	0.1122 (12)	0.410 (3)	0.069 (6)*	

### Atomic displacement parameters (Å^2^)

**Table:**

	*U*^11^	*U*^22^	*U*^33^	*U*^12^	*U*^13^	*U*^23^
S1	0.0880 (4)	0.0664 (3)	0.0596 (3)	−0.0262 (2)	0.0302 (2)	0.00300 (19)
N1	0.0597 (7)	0.0492 (7)	0.0585 (8)	0.0007 (5)	0.0303 (6)	0.0018 (5)
N2	0.0642 (8)	0.0480 (7)	0.0573 (8)	−0.0075 (6)	0.0275 (7)	0.0007 (6)
N3	0.0670 (8)	0.0561 (7)	0.0596 (8)	−0.0104 (6)	0.0315 (7)	−0.0027 (6)
C1	0.0754 (11)	0.0668 (10)	0.0659 (11)	−0.0117 (8)	0.0349 (9)	−0.0038 (8)
C2	0.0902 (14)	0.0930 (14)	0.0586 (11)	−0.0119 (11)	0.0317 (10)	−0.0073 (10)
C3	0.0928 (14)	0.0919 (14)	0.0675 (12)	0.0027 (11)	0.0476 (11)	0.0111 (10)
C4	0.0926 (14)	0.0691 (11)	0.0825 (13)	−0.0089 (10)	0.0527 (12)	0.0085 (9)
C5	0.0784 (11)	0.0588 (9)	0.0701 (11)	−0.0105 (8)	0.0401 (9)	−0.0037 (8)
C6	0.0627 (9)	0.0510 (8)	0.0620 (9)	0.0003 (6)	0.0352 (8)	−0.0008 (7)
C7	0.0656 (9)	0.0496 (8)	0.0593 (9)	−0.0042 (7)	0.0317 (7)	−0.0034 (7)
C8	0.0561 (8)	0.0444 (7)	0.0634 (9)	0.0003 (6)	0.0309 (7)	0.0010 (6)
C9	0.0535 (8)	0.0537 (8)	0.0560 (8)	−0.0063 (6)	0.0257 (7)	−0.0044 (6)
C10	0.0799 (13)	0.0721 (12)	0.1209 (18)	−0.0164 (10)	0.0643 (13)	−0.0325 (12)
C11	0.0858 (14)	0.1098 (17)	0.1139 (18)	−0.0380 (13)	0.0699 (14)	−0.0354 (14)
C12	0.0735 (11)	0.0781 (12)	0.0649 (11)	−0.0267 (9)	0.0242 (9)	−0.0005 (9)
C13	0.0830 (13)	0.0539 (10)	0.0972 (15)	−0.0054 (9)	0.0387 (11)	−0.0048 (9)
C14	0.0668 (10)	0.0599 (10)	0.0840 (12)	−0.0036 (8)	0.0427 (9)	−0.0096 (8)
C15	0.123 (2)	0.117 (2)	0.1007 (19)	−0.0629 (18)	0.0376 (16)	0.0096 (15)

### Geometric parameters (Å, °)

**Table:**

S1—C8	1.6776 (17)	C5—H5	0.9300
N1—C7	1.276 (2)	C6—C7	1.459 (2)
N1—N2	1.367 (2)	C7—H7	0.9300
N2—C8	1.346 (2)	C9—C14	1.366 (2)
N2—H2	0.88 (2)	C9—C10	1.368 (3)
N3—C8	1.336 (2)	C10—C11	1.378 (3)
N3—C9	1.432 (2)	C10—H10	0.9300
N3—H3	0.90 (2)	C11—C12	1.368 (3)
C1—C2	1.371 (3)	C11—H11	0.9300
C1—C6	1.386 (3)	C12—C13	1.370 (3)
C1—H1	0.9300	C12—C15	1.515 (3)
C2—C3	1.382 (3)	C13—C14	1.381 (3)
C2—H2A	0.9300	C13—H13	0.9300
C3—C4	1.374 (3)	C14—H14	0.9300
C3—H3A	0.9300	C15—H15B	0.9600
C4—C5	1.376 (3)	C15—H15C	0.9600
C4—H4	0.9300	C15—H15A	0.9600
C5—C6	1.391 (2)		
			
C7—N1—N2	115.46 (14)	N3—C8—N2	116.54 (15)
C8—N2—N1	120.89 (14)	N3—C8—S1	124.09 (12)
C8—N2—H2	118.7 (13)	N2—C8—S1	119.37 (13)
N1—N2—H2	120.4 (13)	C14—C9—C10	119.28 (16)
C8—N3—C9	123.97 (14)	C14—C9—N3	119.92 (15)
C8—N3—H3	117.3 (13)	C10—C9—N3	120.80 (15)
C9—N3—H3	118.6 (13)	C9—C10—C11	120.03 (18)
C2—C1—C6	120.59 (18)	C9—C10—H10	120.0
C2—C1—H1	119.7	C11—C10—H10	120.0
C6—C1—H1	119.7	C12—C11—C10	121.56 (19)
C1—C2—C3	120.49 (19)	C12—C11—H11	119.2
C1—C2—H2A	119.8	C10—C11—H11	119.2
C3—C2—H2A	119.8	C11—C12—C13	117.60 (18)
C4—C3—C2	119.36 (18)	C11—C12—C15	120.8 (2)
C4—C3—H3A	120.3	C13—C12—C15	121.6 (2)
C2—C3—H3A	120.3	C12—C13—C14	121.51 (19)
C3—C4—C5	120.53 (18)	C12—C13—H13	119.2
C3—C4—H4	119.7	C14—C13—H13	119.2
C5—C4—H4	119.7	C9—C14—C13	119.93 (17)
C4—C5—C6	120.38 (18)	C9—C14—H14	120.0
C4—C5—H5	119.8	C13—C14—H14	120.0
C6—C5—H5	119.8	C12—C15—H15B	109.5
C1—C6—C5	118.64 (16)	C12—C15—H15C	109.5
C1—C6—C7	121.86 (15)	H15B—C15—H15C	109.5
C5—C6—C7	119.48 (15)	C12—C15—H15A	109.5
N1—C7—C6	122.22 (15)	H15B—C15—H15A	109.5
N1—C7—H7	118.9	H15C—C15—H15A	109.5
C6—C7—H7	118.9		
			
C7—N1—N2—C8	179.41 (15)	N1—N2—C8—N3	0.1 (2)
C6—C1—C2—C3	0.4 (3)	N1—N2—C8—S1	−179.63 (11)
C1—C2—C3—C4	−0.9 (4)	C8—N3—C9—C14	113.67 (19)
C2—C3—C4—C5	0.6 (3)	C8—N3—C9—C10	−67.3 (2)
C3—C4—C5—C6	0.2 (3)	C14—C9—C10—C11	−2.5 (3)
C2—C1—C6—C5	0.4 (3)	N3—C9—C10—C11	178.4 (2)
C2—C1—C6—C7	178.92 (18)	C9—C10—C11—C12	0.7 (4)
C4—C5—C6—C1	−0.7 (3)	C10—C11—C12—C13	2.0 (4)
C4—C5—C6—C7	−179.28 (17)	C10—C11—C12—C15	−178.9 (2)
N2—N1—C7—C6	−178.88 (14)	C11—C12—C13—C14	−3.0 (3)
C1—C6—C7—N1	4.7 (3)	C15—C12—C13—C14	177.9 (2)
C5—C6—C7—N1	−176.80 (16)	C10—C9—C14—C13	1.6 (3)
C9—N3—C8—N2	179.12 (14)	N3—C9—C14—C13	−179.34 (18)
C9—N3—C8—S1	−1.2 (2)	C12—C13—C14—C9	1.2 (3)

### Hydrogen-bond geometry (Å, °)

**Table:**

*D*—H···*A*	*D*—H	H···*A*	*D*···*A*	*D*—H···*A*
N2—H2···S1^i^	0.88 (2)	2.48 (2)	3.3522 (15)	170.3 (17)

Symmetry codes: (i) −*x*, −*y*, −*z*.
